# Allergy immunotherapy prescribing trends for grass pollen-induced allergic rhinitis in Germany: a retrospective cohort analysis

**DOI:** 10.1186/s13223-015-0085-x

**Published:** 2015-06-10

**Authors:** Amanda L. McDonell, Ulrich Wahn, Dirk Demuth, Catrina Richards, Charlie Hawes, Jakob Nørgaard Andreasen, Felicia Allen-Ramey

**Affiliations:** Real-World Evidence Solutions and Health Economics & Outcomes Research, IMS Health, 210 Pentonville Road, London, N1 9JY UK; Department for Pediatric Pneumology and Immunology, Charité Medical University, Berlin, Germany; Worked at IMS Health at time of study, Real-World Evidence Solutions and Health Economics & Outcomes Research, London, UK; Global Market Access, ALK-Abelló A/S, Hørsholm, Denmark; Global Health Outcomes, Merck & Co., West Point, PA 19486 USA

**Keywords:** Allergens, Clinical immunology, Paediatrics, Grass pollen allergy, Rhinitis

## Abstract

**Background:**

Allergy immunotherapy is an effective treatment for patients with allergic rhinitis whose symptoms are unresolved with pharmacotherapy. Allergy immunotherapy for grass pollen-induced allergic rhinitis is available in three modalities: subcutaneous immunotherapy and sublingual immunotherapy as a tablet or drop. This study aimed to understand trends in allergy immunotherapy prescribing and practice patterns for grass allergies in adult and paediatric patients in Germany.

**Methods:**

A retrospective cohort study was conducted using IMS Disease Analyzer in Germany. Patients with an allergy immunotherapy prescription for grass pollen (Anatomical Therapeutic Chemical [ATC] classification code V01AA02) from September 2005 to December 2012 were included in the study. General Practitioners (GPs), dermatologists, Ear, Nose and Throat (ENT)-specialists, paediatricians and pneumologists were included as the allergy immunotherapy prescribing physicians in the study. Descriptive analyses were conducted on patient characteristics at index and prescribing physician specialty; a test for trend was conducted for timing of initiation of first allergy immunotherapy prescription in each annual prescribing season.

**Results:**

Eighteen thousand eight hundred fifty eligible patients were identified during the study period. The majority of patients received subcutaneous immunotherapy; however, the proportion of patients receiving sublingual immunotherapy tablets increased from 8 % in 2006/2007 to 29 % in 2011/2012 (*p* < 0.001). Initiation of subcutaneous immunotherapy and Oralair® generally peaked during each prescribing year in two seasons (September-October and January) while GRAZAX® prescriptions peaked in autumn (September-October). ENT-specialists and dermatologists were the largest allergy immunotherapy prescribers in adults, while paediatricians and ENT-specialists were the largest prescribers of allergy immunotherapy in paediatric patients.

**Conclusions:**

Subcutaneous immunotherapy remained the dominant allergy immunotherapy modality for grass pollen-induced allergic rhinitis in Germany for adult and paediatric patients; however, there was a marked increase in proportion of patients receiving sublingual immunotherapy tablets from 2006/2007 to 2011/2012, after their introduction to the market in 2006. ENT-specialists, dermatologists and paediatricians were responsible for the majority of prescribing. The predominance of particular modalities within certain physician specialties likely reflects different treatment goals or needs.

## Background

Allergic rhinitis (AR) affects 23 % of the population in Western Europe, with approximately half affected by grass pollen-induced rhinitis [1]. Allergy Immunotherapy (AIT) desensitizes the immune system to allergens (including grass pollen) and is a treatment option to alleviate AR symptoms when a patient is not adequately managed with symptomatic medication. The benefits of AIT can include symptom reduction, reduced need for symptom relieving medications, disease modification, improvement in asthma symptoms, and prevention of new sensitizations and allergic asthma [2–13]. AIT is available in three modalities: subcutaneous immunotherapy (SCIT) and sublingual immunotherapy (SLIT) in both a tablet (SLIT-tablet) and drop (SLIT-drop) formulation. SLIT-tablet is a relatively new modality, with two products (GRAZAX®: standardized allergen extract of grass pollen from *Phleum pratense* 75,000 Standardized Quality units Tablet per oral lyophilisate; and Oralair®: 5-grass pollen allergen extract tablet, 100 index of reactivity [IR] & 300 IR) available in European countries since 2006 and 2008, respectively [14, 15].

AIT is effective and well tolerated in adults and children, and the Joint Task Force on Practice Parameters recommends that AIT can be considered for patients who have demonstrable evidence of specific immunoglobulin E antibodies to clinically relevant allergens [16]. Although the comparative efficacy of SCIT and SLIT has not been established with direct comparative randomized controlled trials, the clinical efficacy of SLIT (irrespective of allergen) has been suggested to be similar to SCIT based on select open, controlled trials in an evidence review [17]; however, a review of meta-analyses has suggested greater efficacy of SCIT [18], while other studies have found no statistically significant difference between modalities [19].

In practice, choice of modality is likely based on patient and prescriber preferences. SCIT allows more time for patient-physician interaction as it is physician-administered and can treat multiple allergens at once; however, the treatment regimen is lengthy (generally 3–5 years), the frequency of visits may be inconvenient, and the modality unattractive to patients with needle phobia [20]. Although speculative, given that SLIT-tablets have not been available in the United States (US) until recently, SCIT may be found to be preferred amongst allergists in the US as these physicians typically formulate extracts themselves; whereas in Europe virtually all AIT is formulated by extract manufacturers [21].

The three AIT modalities (SCIT, SLIT-drops, SLIT-tablets) have been available for several years in Germany, with treatment initiated by a range of physician specialties, some with sub-specialty in allergology. In the present study we describe the AIT prescribing and practice patterns for paediatric and adult grass pollen-induced AR patients in Germany. The study covers the period pre- and post-introduction of SLIT tablets, thus enabling the study of the impact of this new modality on prescribing patterns.

## Methods

### Study design and data source

This was a retrospective cohort study using the IMS Disease Analyzer (DA) database in Germany. DA collects data from Electronic Medical Records of General Practitioners (GP) and office-based specialists and has been shown to be representative of the German population [22]. GPs, dermatologists, Ear, Nose and Throat (ENT)-specialists, paediatricians, and pneumologists were included in this study. Dermatologists were included based on their history as the original allergists in Germany and paediatricians based on the frequency of allergology training in this specialty. Allergology is a sub-specialty in Germany, rather than a distinct specialty (e.g., paediatricians can have sub-specialty in allergology); this is in contrast to some countries where allergology is a distinct specialty (e.g., US).

### Patient inclusion criteria

Patients were included in the study if they received an AIT prescription with an Anatomical Therapeutic Chemical (ATC) classification code for grass pollen (V01AA02) during the study period. Patients were excluded if they had multiple AIT modalities prescribed at index or had a record of an insect allergy (ATC V01AA07), since insect venom AIT regimens differ from that of seasonal allergies. There were no criteria for inclusion or exclusion of patients receiving multi-allergen immunotherapy.

### Analyses

To meet the study objectives, two specific analyses were conducted. The first was a cross-sectional analysis to determine temporal trends of AIT during each “prescribing year” (1 September – 31 August annually) from 2005 to 2012. The prescribing year was designated to begin in September based on clinical input regarding customary approach to AIT initiation in Germany, as prescriptions started in September are intended to alleviate symptoms in the upcoming season (co-author: U. Wahn). The same patient could have been included in analyses across years, but only their first prescription each year was included in annual analyses. Patients were grouped by modality with SLIT-tablets segmented into GRAZAX® and Oralair®.

McNemar’s test of difference between first and last year of the study period and Chi-squared test for trend over time were used to determine statistical significance of differences in proportion of patients prescribed each modality between years. Initial and total AIT prescriptions were compared within each year to observe differences in trend that may have arisen from specification of the prescribing year.

A second analysis was undertaken to examine patient characteristics and physician specialities related to AIT. The first AIT prescription identified for a patient between September 1st 2005 and December 31st 2012 was considered the index prescription. Medical records prior to index were searched for prior AIT use. Information on the physician associated with the index prescription determined physician specialty. The proportion of patients prescribed each modality by physician type and age group was calculated over the entire study period and in the calendar year of 2012 alone representing the most recent year. Descriptive statistics for number of AIT patients treated by each prescribing physician were also calculated.

Patients were characterized at index by age, gender, co-morbidities, history of grass pollen AIT use, and insurance status. Paediatric patients were <18 years of age at index. A diagnosis code of a relevant co-morbidity (asthma, sinusitis, atopic dermatitis, conjunctivitis, urticaria, other documented allergies [via ATC code]) or prescription of an asthma or atopic dermatitis medication pre-index were used to determine co-morbidities. Patients were classified as publicly or privately insured. Prescriptions of concomitant allergic rhinoconjunctivitis (ARC) medication were based on record of a prescription one month pre-index or during the entire post-index period. The length of the post-index period varied between patients and ended when a patient no longer attended a physician in DA or on December 31, 2012.

## Results

### Patient characteristics

A total of 18,850 patients were prescribed AIT for grass pollen allergy during the study period. Patient characteristics are summarized in Table 1. Age of patients was consistent between AIT modalities, with a mean age of 12.1 years and 34.3 years in the paediatric and adult populations, respectively. In the paediatric population 63 % of patients were male. 88 % of patients were publicly insured. Five percent of patients had a record of a grass-related AIT prescription prior to index.

**Table 1:** Patient characteristics at allergy immunotherapy initiation

Characteristic	All patients (*n* = 18,850)	<18 years of age (*n* = 6425)	≥18 years of age (*n* = 12,425)
Age^a^	26.7 ± 14.35 (24.0)	12.1 ± 3.24 (12.0)	34.3 ± 11.78 (33.0)
SLIT-tablets	28.5 ± 14.76 (27.0)	12.0 ± 3.31 (12.0)	35.2 ± 12.15 (34.0)
SLIT-drop	26.5 ± 15.44 (25.0)	11.1 ± 3.41 (11.0)	35.9 ± 11.97 (35.0)
SCIT	26.2 ± 14.09 (24.0)	12.2 ± 3.20 (12.0)	33.9 ± 11.61 (32.0)
Gender, male	53 %	63 %	48 %
SLIT-tablets	51 %	61 %	48 %
SLIT-drop	53 %	65 %	46 %
SCIT	54 %	64 %	48 %
Health insurance			
State (public)	16,502 (88 %)	5729 (89 %)	10,773 (87 %)
Private	2348 (12 %)	696 (11 %)	1652 (13 %)
Prior AIT usage at index	957 (5 %)	351 (5 %)	606 (5 %)
SLIT-tablets	31 (1 %)	4 (<1 %)	27 (1 %)
SLIT-drop	35 (4 %)	11 (3 %)	24 (4 %)
SCIT	891 (7 %)	336 (7 %)	555 (6 %)
Co-morbidities^b^			
Asthma	7904 (42 %)	2967 (46 %)	4937 (40 %)
Conjunctivitis	4215 (22 %)	1709 (27 %)	2506 (20 %)
Atopic Dermatitis	3770 (20 %)	1634 (25 %)	2136 (17 %)
Sinusitis	2954 (16 %)	859 (13 %)	2095 (17 %)
Concomitant ARC usage^c^	13,403 (71 %)	5053 (79 %)	8350 (67 %)

^a^Mean ± SD (median)

^b^Co-morbidities not mutually exclusive

^c^At index or post-index

The most common co-morbidities observed in AIT patients at index were asthma (42 %), conjunctivitis (22 %), atopic dermatitis (20 %), and sinusitis (16 %). Co-morbidities were not mutually exclusive. Co-morbidities of other allergies and urticaria occurred in less than 15 % of patients. The percentage of patients with a co-morbidity was largely consistent across AIT modalities (77 % SLIT-tablet, 81 % SLIT drop, 79 % SCIT). The majority (71 %) of patients had a record of concomitant ARC medication.

### Prescribing trends by AIT modality

**Fig. 1:**
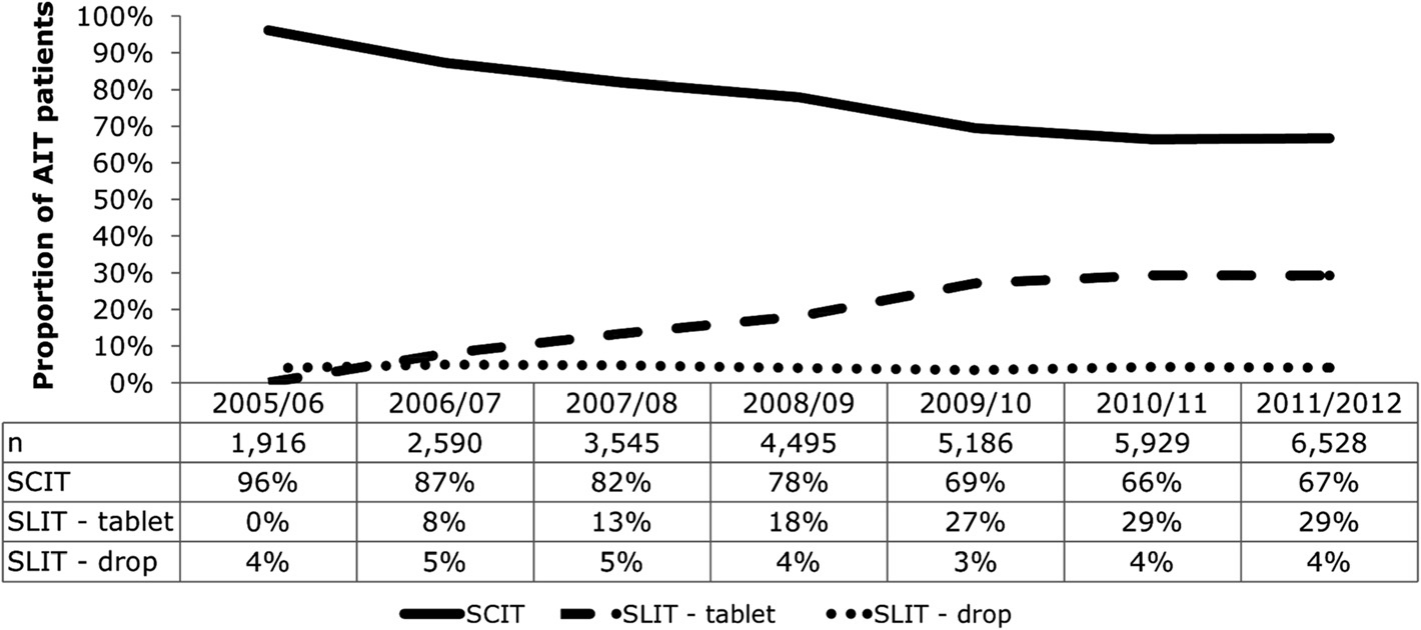
First allergy immunotherapy prescription in each prescribing year, by modality. SCIT was the primary modality prescribed throughout the study period; however, there was a statistically significant increase in the proportion of patients treated with SLIT-tablets from 8 % in 2006/2007 to 29 % in 2011/2012 (*p* < 0.001, McNemar’s test). The proportion of patients prescribed SLIT-drops was marginal at 3–5 % of total prescribing each year

SCIT was the primary modality prescribed throughout the study period; however, there was a statistically significant increase in the proportion of patients treated with SLIT-tablets from 8 % in 2006/2007 to 29 % in 2011/2012 (*p* < 0.001, McNemar’s test) (Fig. 1). The increase in SLIT-tablets between each prescribing year was also statistically significant (*p* < 0.001, Chi-squared test for trend). Following SLIT-tablet approval in the paediatric population in 2008, its share of prescribing increased from 9 % in 2008/09 to 26 % in 2011/12. The proportion of patients prescribed SLIT-drops was marginal at 3–5 % of total prescribing each year; given this low usage, the results and discussion will focus on SCIT and SLIT–tablet modalities. Note: the sample of physicians increased throughout the study period (see “Characteristics of AIT prescribers” below), therefore the conclusion cannot be drawn that the number of patients treated with AIT increased.

### Prescribing trends by allergy season

**Fig. 2:**
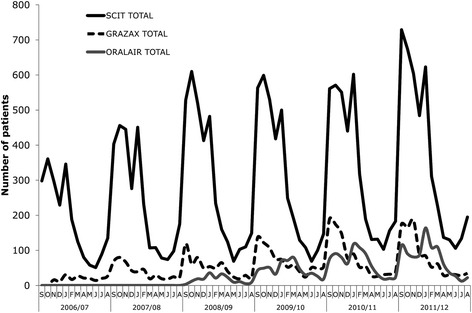
Initiation of allergy immunotherapy, by prescribing season. Initiation of SLIT-tablet and SCIT within the prescribing year generally had two peaks: autumn and the January–April period. GRAZAX® had a clear annual peak in September–November, while Oralair® had a peak in initiations from January to March of each prescribing year; however, a second peak indicated a proportion of patients (27 % on average) initiated Oralair® from September to November. SCIT prescriptions exhibited a biphasic trend, with initial prescriptions peaking from September to October and in January

Initiation of SLIT-tablet and SCIT within each prescribing year generally had two peaks: autumn and the January–April period (Fig. 2). GRAZAX® had a clear annual peak in September–November, while Oralair® had a peak in initiations from January to March of each prescribing year; however, a second peak indicated a proportion of patients (27 % on average) initiated Oralair® from September to November. SCIT prescriptions exhibited a biphasic trend, with initial prescriptions peaking from September to October and in January. In the analysis of total prescriptions (not shown graphically) initial and total SCIT and Oralair® prescriptions tracked each other closely. Total GRAZAX® prescriptions oscillated throughout the year, indicative of its indication for year-round treatment. Less than 10 % of all patients initiated treatment in-season (May–July).

### Characteristics of AIT prescribers

**Fig. 3:**
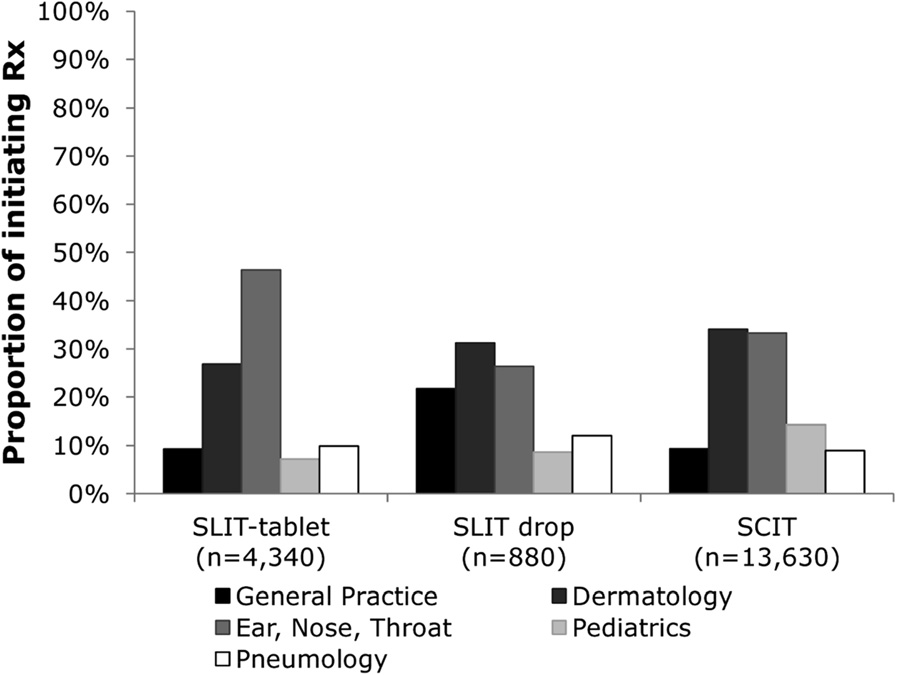
Allergy immunotherapy prescribing for total population, by modality and physician specialty. AIT was prescribed by GPs, dermatologists, ENT-specialists, pediatricians and pneumologists. ENT-specialists and dermatologists together accounted for 67 % of SCIT prescribing, while ENT-specialists accounted for 46 % of SLIT-tablet prescribing

AIT was prescribed by GPs, dermatologists, ENT-specialists, pediatricians and pneumologists (Fig. 3). The DA panel increased over the study period, with the number of physicians prescribing AIT increasing from 239 (2005) to 648 (2012). ENT-specialists and dermatologists together accounted for 67 % of SCIT prescribing, while ENT-specialists accounted for 46 % of SLIT-tablet prescribing.

Over the study period 756 unique physicians prescribed AIT to adult patients. These physicians treated a mean (SD) of 16.4 (30.5) adult AIT patients each. ENT-specialists were the largest prescribers of SLIT-tablets to adults (48 % of SLIT-tablet prescriptions). SCIT was predominantly prescribed by dermatologists and ENT-specialists, representing 42 % and 37 % of SCIT prescriptions, respectively. In 2012 ENT-specialists remained the largest prescribers of SLIT-tablets (50 % of prescriptions), while ENT-specialists and dermatologists together accounted for 79 % of SCIT prescriptions.

A total of 617 unique physicians prescribed AIT to paediatric patients over the study period. These physicians treated a mean (SD) of 10.4 (16.3) paediatric AIT patients each. The largest prescribers of SLIT-tablet were ENT-specialists (43 % of SLIT-tablet prescriptions) while the largest prescribers of SCIT were paediatricians (39 %). In 2012 SLIT-tablets were prescribed largely by ENT-specialists (50 %), while paediatricians accounted for 43 % of SCIT prescriptions.

## Discussion

### Main findings

This retrospective cohort study of patients treated with AIT for grass pollen-induced AR in Germany uniquely examined timing of initiation of treatment and prescribing trends over time. SCIT was the most commonly prescribed AIT modality, although the proportion of patients receiving SLIT-tablet increased markedly following launch in 2006. SLIT-drops had low utilization with a share of 3–5 % of the total annual AIT prescribing across the study period. Initiation of SLIT-tablets GRAZAX® and Oralair® generally occurred in the autumn and January–April period, respectively, with a large proportion of Oralair® patients also receiving a prescription in autumn. SCIT had a biphasic trend, with prescriptions peaking in autumn and January. ENT-specialists and dermatologists were the largest AIT prescribers overall. The most common co-morbidities observed in patients at index were asthma, conjunctivitis, atopic dermatitis, and sinusitis.

### Our findings in context

This is the first study of AIT prescribing trends in clinical practice that examines all available treatment modalities. Other studies have estimated the use of AIT among AR patients or focused on a specific route of AIT. Cross-sectional survey data and medical record review of seasonal AR patients in a small German sample indicated 35 % of children/adolescents and 65 % of adults received AIT [23], while claims data from 2007 to 2010 in Germany found 7 % of patients with AR received AIT [24]. A previous publication reported that approximately 25 % of AIT patients in Germany were treated with SLIT-tablets or SLIT-drops [25], whereas our study found that 33 % of patients received SLIT-tablets or SLIT-drops (2011/2012 prescribing year). Biermann et al. [24] reported proportions of AR patients with co-morbid asthma ranging from 16.0% to 17.4 % from 2007 to 2010 while asthma patients with co-morbid AR ranged from 47.3 to 48.1 %. The results of our study yielded estimates of co-morbid asthma of 40 % in adult patients and 46 % in paediatric patients prior to AIT initiation.

### Clinical relevance

AIT is intended to induce immunologic tolerance hence pre-seasonal initiation for grass pollen allergies is ideal to minimize symptoms experienced during the season. The decision of when to initiate AIT should therefore be driven primarily by knowledge of timing of the grass pollen season. There was evidence that a large proportion of SLIT-tablet patients initiated treatment in the February–April time period, rather than four months in advance of the typical May-onset season as ideally recommended for optimal benefit. Furthermore, there was evidence that Oralair®, a product recommended for use only until the end of the season, is initiated in the September–November period by an average of 27 % of patients. While these patterns may reflect clinical experience surrounding the ability of patients to achieve adequate benefit with a shorter lead time (e.g., 12 weeks prior) or full treatment benefit with a greater lead time (e.g., prior autumn season), these differences likely impact the effectiveness and estimated cost of treatment.

The results also highlight insights on age of patients receiving AIT and type of physicians prescribing each modality. Given that AIT has been found to be well-tolerated and effective in paediatric patients [16], it is interesting that only one–third of patients in the study sample were <18 years of age. This may indicate a need for education on proven benefit of AIT in paediatric patients, particularly given evidence that initiation of AIT in childhood may have additional benefits of preventing the onset of new sensitizations and asthma [2–10].

In terms of prescribing physician trends it is likely that physician preferences impact choice of modality. In the paediatric population paediatricians were the largest prescribers of SCIT, perhaps indicating a preference for this more established modality; whereas in both adult and paediatric patients ENT-specialists were the largest SLIT-tablet prescribers, possibly as it is more convenient to administer. In markets where allergy specialists administer AIT, the introduction of tablets may present greater complexity due to the need to consider multiple treatment options and patient characteristics suited to a particular modality. For other specialities, the oral SLIT-tablet may be viewed as a simpler approach to administering AIT. Furthermore, there may be economic incentives for SCIT due to the need for visits for injection. While country-by-country variation in reimbursement, available modalities, and specialty types may impact prescribing trends, our findings can still be of interest to other markets, particularly as different physician specialties determine how to integrate new treatment modalities into their practice.

### Strengths & limitations

The primary strength of this study was the longitudinal examination of AIT prescriptions written for management of grass pollen-induced AR patients in clinical practice for a large sample of patients. The study also spanned the period pre- and post-SLIT-tablet launch, providing insight on the impact of this new modality on prescribing dynamics. Given the lack of other publications of real-world AIT usage, these findings contribute to the body of evidence of AIT practice.

Limitations of this study were the inability to link patients between physicians, meaning a patient’s record included only the treatment provided by a single physician, and the reliance on physicians to accurately record information within the electronic medical records. Results may also have been impacted by the end of the German special allergy budget which changed reimbursement for administration of AIT during the study period.

## Conclusions

As the first analysis of AIT prescribing trends in grass pollen-induced AR patients, this study highlighted the differences in onset of treatment initiation for different modalities and trends in prescribing by physician specialty. SCIT remained the dominant AIT modality in Germany, despite strong uptake of SLIT-tablets. Initiation of SCIT and Oralair® generally peaked in two seasons while GRAZAX® prescriptions peaked in autumn. ENT-specialists were the largest prescribers of SLIT-tablets, while paediatricians, dermatologists, and ENT-specialists were the largest SCIT prescribers. The study spanned a period where the treatment paradigm adapted to the introduction of the SLIT-tablet modality, with results showing predominance of particular modalities within certain physician specialties likely based on different treatment goals or needs. A possible reduction in the onset of future sensitizations and asthma through increased initiation of AIT in paediatric patients is an additional benefit that warrants further consideration. While the results are most relevant to clinical practice in Germany, they may reflect patterns in other countries with similar treatment paradigms.

## Abbreviations

AIT: Allergy immunotherapy
AR: Allergic rhinitis
ARC: Allergic rhinoconjunctivitis
ATC: Anatomical Therapeutic Chemical
DA: Disease Analyzer
ENT: Ear, Nose and Throat
GP: General Practitioner
SCIT: Subcutaneous immunotherapy
SLIT: Sublingual immunotherapy
